# Feasibility Study of Modified Underwater Endoscopic Mucosal Resection for Colorectal Polyps

**DOI:** 10.1002/deo2.70294

**Published:** 2026-02-14

**Authors:** Kaizo Kagemoto, Koichi Okamoto, Yoji Takeuchi, Yasuyuki Okada, Motoko Sei, Shota Fujimoto, Ryo Shinomiya, Takeshi Mitsuhashi, Takanori Yoshimoto, Reiko Yokoyama, Tomoyuki Kawaguchi, Yoshifumi Kida, Yasuhiro Mitsui, Yutaka Kawano, Masahiro Sogabe, Hiroshi Miyamoto, Yasushi Sato, Tetsuji Takayama

**Affiliations:** ^1^ Department of Gastroenterology and Oncology Institute of Biomedical Sciences Tokushima University Graduate School Tokushima Japan; ^2^ Department of Endoscopy and Endoscopic Surgery Gunma University Hospital Gunma Japan

**Keywords:** colorectal polyps, modified UEMR, submucosal tissue thickness, underwater, UEMR

## Abstract

**Objectives:**

Underwater endoscopic mucosal resection (UEMR) is widely performed for colorectal tumors. However, in our experience, it is sometimes difficult to keep a clear endoscopic view underwater, due to dirty fluid inflow or insufficient water pooling after grasping the polyp, or bleeding just after endoscopic resection. To compensate for such challenges, we reported modified UEMR (M‐UEMR) as a procedure for snaring underwater and undergas resection. Therefore, we conducted a prospective clinical trial to investigate the safety and efficacy of M‐UEMR.

**Methods:**

This single‐center prospective study was conducted at Tokushima University Hospital. Patients with 10–25 mm colorectal polyps were enrolled. The polyps were snared underwater, then infused water was removed, and the lumen was inflated with CO_2_ for resection, as described in our previous report. Measured outcomes were R0 resection rate and adverse events such as bleeding, perforation, and post‐polypectomy syndrome. In addition, we evaluated the en bloc resection rate and thickness of submucosal (SM) tissue of the resected specimens.

**Results:**

Forty patients were enrolled, and the R0 resection rate was 80% (95% confidence interval [CI]: 64.4–90.9). No procedure‐related adverse events were observed. En bloc resection was 92.5% (95% CI: 79.6–98.4). The median thickness of SM tissue (range) was 574 µm (241–2632) at the center of the specimen.

**Conclusion:**

M‐UEMR demonstrated a high R0 resection rate with a safe profile. M‐UEMR is expected to be utilized as an alternative technique to UEMR for colorectal polyps in patients with difficulty maintaining a clear visual field.

## 1 Introduction

Colorectal cancer (CRC) is the third most common cancer and the second leading cause of cancer death worldwide, and is on the rise [1]. Endoscopic resection of colorectal polyps has been shown to reduce colorectal cancer‐related mortality, and polyps can be removed with a variety of treatment techniques [2]. Endoscopic mucosal resection (EMR) has been widely performed for the resection of colorectal polyps, a procedure that requires submucosal injection [3, 4]. Underwater EMR (UEMR) was first reported by Binmoeller et al. as a simple procedure performed underwater without submucosal injection [5]. Recently, the usefulness and safety of UEMR have been demonstrated [6, 7]. In a randomized controlled trial, UEMR was significantly superior to conventional EMR in terms of R0 resection rate for intermediate‐size sessile colorectal polyps [8]. Based on these results, UEMR is becoming a popular alternative procedure to conventional EMR. On the other hand, even after successful capturing underwater, we sometimes encounter situations in which it is difficult to keep a clear endoscopic view underwater, such as with dirty fluid inflow or insufficient water pooling after grasping the polyp, or bleeding just after endoscopic resection. In addition, there are some concerns about aggravation of peritonitis due to water leakage in cases of perforation [9]. Therefore, we reported modified UEMR (M‐UEMR) as an alternative procedure for cases unfavorable for conventional UEMR [10]. M‐UEMR involves underwater snaring followed by under gas resection with electrocautery. The key advantage of UEMR is the ease of snaring underwater, and M‐UEMR can also theoretically be performed while taking advantage of this strength. Although M‐UEMR can resolve the concerns mentioned above, the heat‐sink effect of UEMR would be lost. Given the significant heat‐sink effect demonstrated by Tseng et al. in a porcine colon model [11], careful evaluation of adverse events such as delayed bleeding and post‐polypectomy syndrome is warranted [5, 12, 13]. Therefore, we conducted a prospective clinical trial to investigate the safety and efficacy of M‐UEMR.

## 2 Methods

### 2.1 Study Design

This was a single‐center prospective study designed to investigate the safety and efficacy of M‐UEMR for colorectal polyps. The study was conducted at Tokushima University Hospital (Tokushima, Japan) between September 2022 and September 2023. The study protocol was approved by the ethics committee of Tokushima University Hospital. This trial was registered in the University Hospital Medical Information Network Clinical Trials Registry (UMIN 000048503). This study adheres to the STROBE statement to provide clear reporting in observational studies.

### 2.2 Patients

We enrolled the consecutive patients who were referred to our hospital for the treatment of colorectal polyps (adenoma, intramucosal adenocarcinoma). Eligibility criteria included: 1) patient aged 20–80 years old and 2) polyp size of 10–25 mm in diameter. Exclusion criteria included: 1) pedunculated polyp, 2) submucosal (SM) deep invasion cancer, 3) residual polyp after endoscopic resection, 4) inflammatory bowel disease, 5) familial polyposis, 6) electrolyte abnormality, or 7) severe organ failure. Patients receiving an antithrombotic drug were included in this trial, and they were managed based on Japanese gastrointestinal endoscopy guidelines for patients undergoing antithrombotic treatment [14, 15]. Written informed consent was obtained from all patients prior to study participation.

### 2.3 Procedure

**FIGURE 1:**
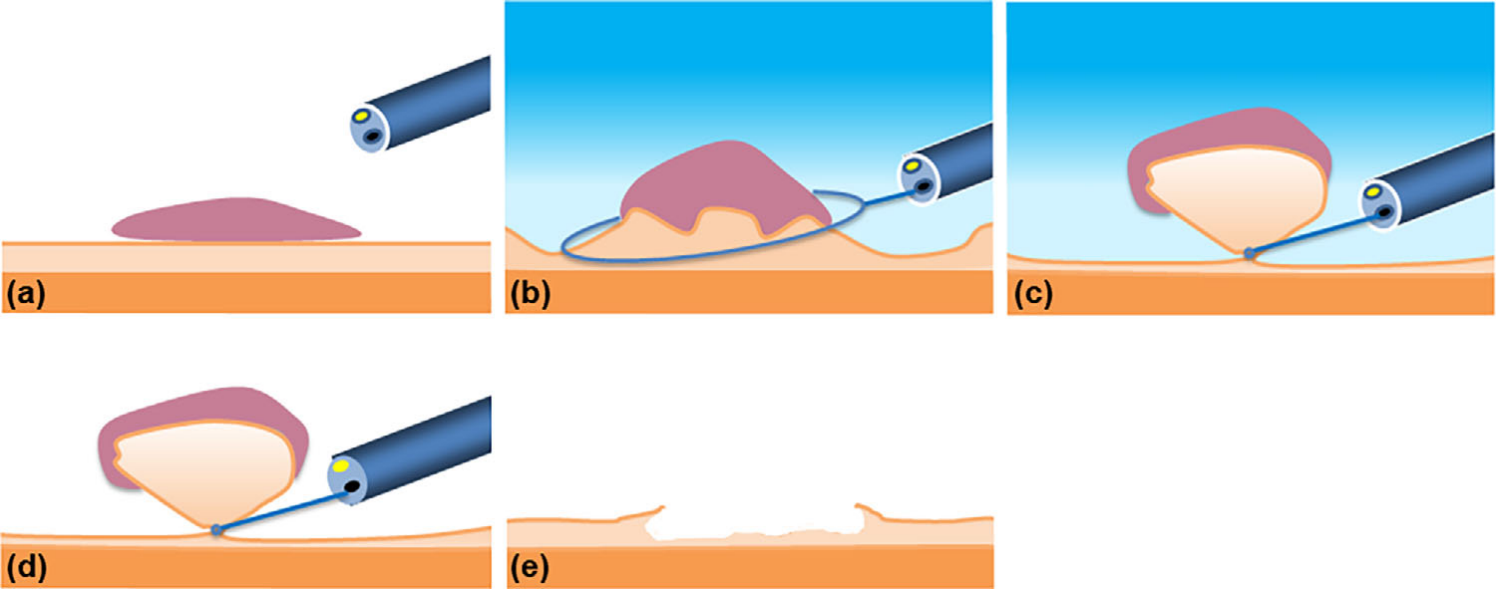
Schematic diagrams of modified underwater endoscopic mucosal resection (M‐UEMR). (a) The target lesion was detected. (b, c) Water immersion and snaring the polyp underwater. (d) Infused water was suctioned and replaced with CO_2_ after snaring. (e) The polyp was resected by electrocautery.

**FIGURE 2:**
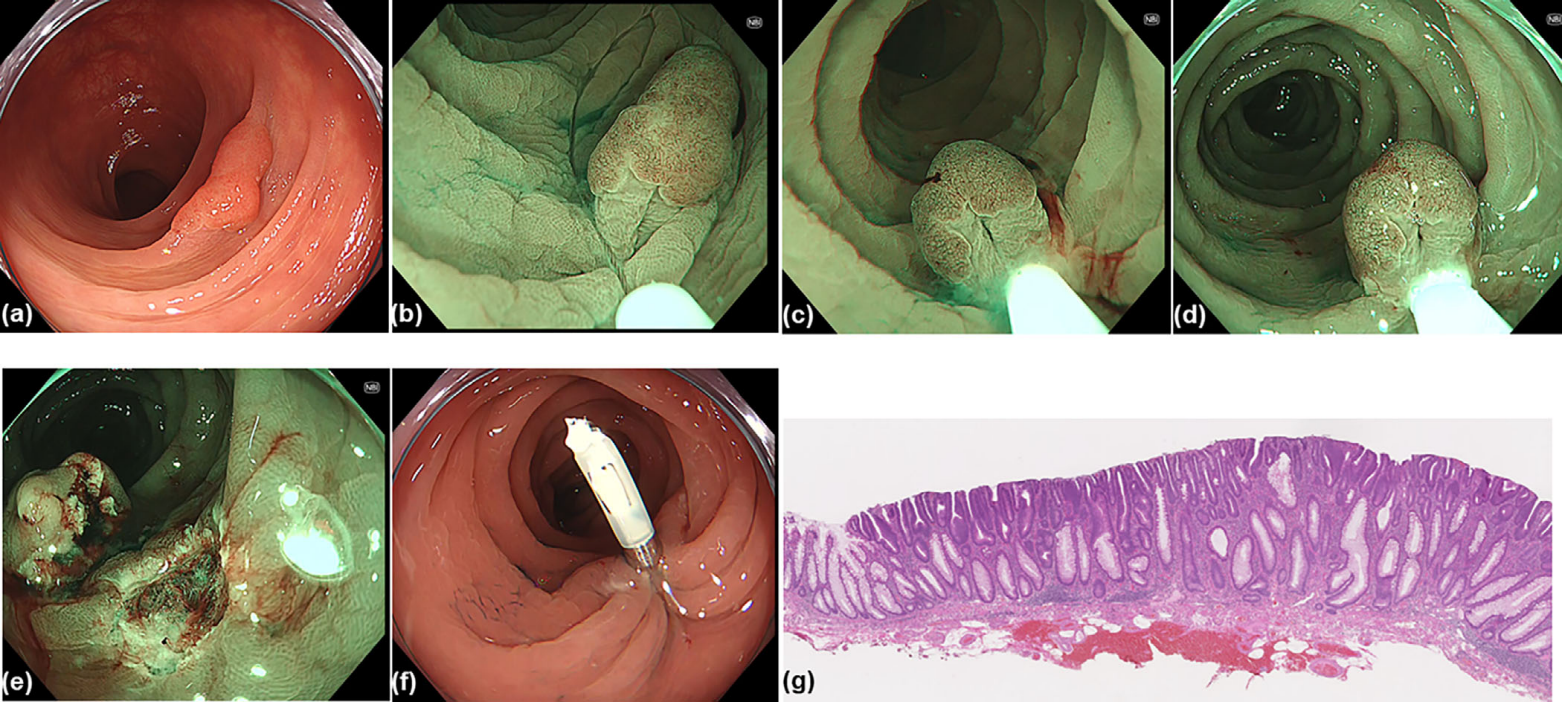
Modified underwater endoscopic mucosal resection for a colorectal polyp. (a) White light imaging (WLI) showed a slightly elevated lesion (0‐IIa) in the transverse colon. (b, c) Narrow band imaging (NBI); underwater snaring of the polyp. (d) Removing the water and inflating with CO_2_ after snaring. (e) The wound had no residual lesion after resection. (f) Clipping with ease. (g) Hematoxylin and eosin (H&E) staining (orig. mag. × 10); a low‐grade adenoma with sufficient submucosal tissue.

All procedures were carried out with a high‐definition video colonoscope (PCF‐H290ZI, CF‐HQ290ZI, and CF‐XZ1200I [Olympus, Tokyo, Japan], or EC‐L600ZP7 [Fujifilm, Tokyo, Japan]). Colonoscopies were performed with CO_2_ insufflation. When the target lesion was detected, the size was measured by comparing it with an open snare and carefully assessed prior to resection. The polyps were observed with image‐enhanced endoscopy (IEE), such as narrow‐band imaging (NBI) or blue laser imaging (BLI), in addition to white light imaging (WLI), and chromoendoscopy if necessary. We carefully assessed lesions inappropriate for endoscopic resection, which were suspected of SM deep invasion, such as lesions with a pattern of Type 3 according to the Japan NBI Expert Team (JNET) classification, or pit patterns of type V according to Kudo's classification [16, 17]. Morphology was classified according to the Paris classification [18]. M‐UEMR procedures were performed as previously described [10]. Schematic diagrams and endoscopic images of M‐UEMR are shown in Figures 1 and 2, respectively. The procedure is also demonstrated in Video S1. First, the colorectal lumen was deflated by suctioning out the air, and then degassed water was infused using a mechanical water pump (OFP‐2; Olympus) until the lumen was filled. The polyp was captured by an electrosurgical snare underwater. A 10, 15, or 25 mm snare (Snaremaster, Snaremaster Plus; Olympus) was used according to polyp size. The procedure up to this point was the same as for conventional UEMR [5]. After the polyp was captured with a snare, the water could be easily removed by suctioning and simultaneously inflating the lumen with CO_2_. This allowed the procedure to be performed in a stable water‐free view, and there was no need to worry about water leakage to the abdominal cavity in case of perforation. After water removal, resection was performed using electrocautery (Endo‐cut I, Effect 2; VIO3, Erbe Elektromedizin GmbH, Tubingen, Germany). The mucosal defect after resection was closed with endoclips (EZ Clip; Olympus). All procedures were performed by four expert endoscopists who were certified by the Japan Gastroenterological Endoscopy Society.

### 2.4 Histological Examination

All retrieved specimens were stretched and pinned onto a rubber plate prior to formalin fixation, sectioned at 2‐mm intervals, and stained with hematoxylin and eosin. Pathological examinations according to the Vienna classification were performed by two pathologists, board‐certified by the Japanese Society of Pathology. The endoscopists and pathologists were consulted to assess whether the specimens contained SM tissue and to measure the thickness from the muscularis mucosa (MM) to the vertical margin (VM) of the resected specimen.

### 2.5 Outcome Measurement

The primary outcome in this study was the R0 resection rate. Secondary outcomes included adverse events, en bloc resection rate, thickness of SM tissue of the resected specimens, and procedure time taken to remove water and replace it with CO_2_. En bloc resection was defined as a lesion resected in a single piece. R0 resection was defined as a single resection where each specimen showed histologically negative margins of the lesion at both resection ends. Adverse events included intraprocedural bleeding, delayed bleeding, perforation, and post‐polypectomy syndrome. Intraprocedural bleeding was defined as bleeding that lasted more than 60 s or required endoscopic intervention just after M‐UEMR. Post‐polypectomy syndrome was defined as abdominal pain, fever (≥37.5°C), leukocytosis, and peritoneal inflammation in the absence of a perforation after endoscopic treatment [19]. Patients who received treatment underwent a medical examination and blood test by a gastroenterologist the following day, and were interviewed regarding the presence or absence of symptoms approximately two weeks later. The thickness of the SM tissue was evaluated at the center and the average of both edges of the specimens on central sections. Procedure time for water replacement with CO_2_ was measured from the polyp captured with a snare to resection after water removal.

### 2.6 Statistical Analysis and Sample Size Calculation

The R0 resection rate and en bloc resection rate with 95% confidence interval (CI) were calculated based on a binomial distribution. In a previous article, the pathological complete resection rate of UEMR for colorectal polyps (10–20 mm) was reported to be 69% [8]. Therefore, we set the expected R0 resection rate of M‐UEMR at 70% and the threshold at 50%, with a power value of 80% and a one‐sided α value of 5%. A sample size of 37 was estimated to be required.

## 3 Results

### 3.1 Baseline Characteristics

Between September 2022 and September 2023, we enrolled 40 patients with 40 polyps. Table 1 shows the clinicopathological characteristics of the patients and polyps. Six patients were taking antithrombotic agents. Twenty‐six (65%) patients were male, with a median age of 50 (range, 35–78) years. The median (range) polyp size was 15 (10–25) mm. The macroscopic types were 0‐IIa (superficial elevated) for 19 polyps and 0‐Is (polypoid) for 21 polyps.

**TABLE 1:** Baseline characteristics of the study subjects.

Patients, *n* = 40	
Male/female, *n*	26/14
Median age, y (range)	50 (35–78)
Use of antithrombotic agent, *n* (%)	6 (15)
**Lesions, *n* = 40**	
Location, *n* (%)	
Cecum	3 (7.5)
Ascending	8 (20)
Transverse	10 (25)
Descending	6 (15)
Sigmoid	8 (20)
Rectum	5 (12.5)
Median size, mm (range)	15 (10–25)
Morphology, *n* (%)	
0‐IIa	19 (47.5)
0‐Is	21 (52.5)

### 3.2 Procedure‐Related Outcomes

The procedure‐related outcomes are shown in Table 2. R0 resection rate, the primary outcome in this trial, was 80% (95% CI: 64.4–90.9). R0, RX, and R1 resections were 32, eight, and zero polyps, respectively. No cases required re‐snaring. No adverse events were observed. En bloc resection was achieved in 37 polyps, and three polyps were resected in two pieces, for a rate of 92.5% (95% CI: 79.6–98.4). The median thickness of SM tissue (range) was 574 µm (241–2632) at the center of specimens and 481 µm (185–2376) at the average of both edges. Median (range) procedure time taken for water removal and replacement with CO_2_ was 9 s (2–42). All lesions were closed with endoclips, with a median of one clip (range, 1–5) used per lesion. Histologically, 37 polyps were adenomas, and three polyps were intramucosal adenocarcinomas.

**TABLE 2:** Procedure‐related outcomes.

R0 resection, *n*	32
Rate, % (95% CI)	80 (64.4–90.9)
R1/RX resection, *n*	0 / 8
Adverse events, *n*	
Intraprocedural bleeding	0
Delayed bleeding	0
Perforation	0
Post‐polypectomy syndrome	0
En bloc resection, *n*	37
Rate, % (95% CI)	92.5 (79.6–98.4)
Thickness of SM tissue, µm, median (range)	
Center of the lesion	574 (241–2632)
Average of both edges	481 (185–2376)
Procedure time taken to remove water, sec, median (range)	9 (2–42)
Histological type, *n* (%)	
Adenoma	37 (92.5)
Intramucosal adenocarcinoma	3 (7.5)

## 4 Discussion

In this study, we first evaluated the efficacy and safety of M‐UEMR for colorectal polyps. The R0 resection rate met the predefined primary endpoint, and no adverse events were observed. In addition, the en bloc resection rate was high, and the resected specimens contained sufficient SM tissue. Therefore, M‐UEMR is expected to become an alternative treatment to conventional UEMR.

UEMR has been reported to be superior to EMR in terms of R0 resection rate, en bloc resection rate, and local recurrence rate in previous reports, which may be due to the ease of snaring by the floating effect and fluid force [8, 20]. In addition, the underwater technique can enhance the sensitivity of endoscopy because of an optical zoom effect and stabilize the endoscopic procedure, even in difficult areas such as the right‐sided colon [5, 21, 22]. These strengths lie in the process preceding snaring, while the so‐called heat‐sink effect, which is not directly supported by clear evidence, may be a strength after snaring. Therefore, we consider that it is not necessary to utilize underwater conditions and that M‐UEMR is a resection method that theoretically takes advantage of the strengths of UEMR. In this study, no cases required re‐snaring. Therefore, the favorable R0 resection rate is attributed to the underwater technique rather than the M‐UEMR. In addition, we consider that the fact that all cases were treated by experts contributed to the outcome. With conventional UEMR, continuing to store water can sometimes be difficult, and we have made efforts to maintain water pooling by providing an additional water supply. However, given the good performance in the current study, it seems that it is not necessary to perform the procedure underwater. It is expected that M‐UEMR can be performed not only in planned situations, but also in incidental situations, such as when water is drained away after snaring polyps during UEMR. However, since M‐UEMR loses the heat‐sink effect, it was necessary to evaluate adverse events such as bleeding, perforation, and post‐polypectomy syndrome. Binmoeller et al. reported that the floating effect allows the lesion to be raised from the muscularis propria, thereby avoiding capture of the deeper layer [5]. Theoretically, the risk of perforation is determined by whether or not the muscle layer is captured during snaring, so the risk of perforation between M‐UEMR and UEMR was considered to be the same. As a result, no cases of perforation or post‐polypectomy syndrome were observed, suggesting the safety of M‐UEMR. In case of perforation during UEMR, water leakage can aggravate peritonitis. Ozeki et al. reported that a longer hospital stay is required in cases of perforation during underwater endoscopic submucosal dissection with moderate to massive fluid collection in the abdominal cavity [23]. With the M‐UEMR procedure, because of water removal, the risk is expected to be reduced when perforation occurs.

UEMR has been reported to be associated with less bleeding than EMR in some articles [24, 25]. There are various hypotheses to explain this finding, one of which is that the lesion becomes polypoid during the underwater procedures, resulting in a smaller wound compared with conventional EMR [8, 12]. M‐UEMR also does not involve submucosal injection, and the associated wound is relatively small, like with UEMR. Therefore, endoscopic clipping could be easily performed as shown in Figure 2. As a result, no intraoperative or postoperative bleeding was observed in this study; however, since there was only a small number of cases, further investigation is needed. In clinical practice, we often experience poor visual field due to intraoperative bleeding after UEMR resection, which can be mostly controlled by snare‐tip coagulation. However, immediate endoscopic management is required, such as water aspiration and stabilizing the field of view. M‐UEMR is a technique that removes water, which is advantageous in such cases. Therefore, lesions that are at high‐risk of bleeding, such as large lesions >20 mm, may be good candidates for M‐UEMR [13]. In addition, since M‐UEMR removes water, it theoretically has the potential to produce more thermal coagulation than UEMR, which may lead to reduced immediate bleeding. However, there is no evidence to support these findings, and it is difficult to accurately evaluate the possibility because of various confounding factors such as the setting of the device, the time of resection, and the type of water used (e.g., saline, sterile water, etc.).

In the present study, the thickness of SM tissue was evaluated in resected specimens. Toyosawa et al. evaluated the resection depth of specimens and reported that SM tissue with UEMR was significantly thicker than with hot snare polypectomy [26]. In a previous study, we evaluated specimens resected by EMR and UEMR and found that the thickness of the SM tissue did not differ significantly between the two groups [27]. These results suggest that UEMR can achieve sufficient VM0 resection in pathological SM1 cancer, like EMR. In the present study, specimens resected by M‐UEMR contained sufficient SM tissue (574 µm) compared with our previous results. Although direct comparison is not appropriate, both studies were performed using stretched specimens before formalin fixation, and, therefore, these results are considered to be generalizable. Based on our results, it is possible that M‐UEMR can also achieve sufficient VM0 resection in pathological SM1 cancer, although further study is needed. The procedure time to remove water may be a disadvantage of M‐UEMR. However, infused water was removed quickly in most cases, with acceptable results. In addition, even in conventional UEMR, water must be aspirated at the end of the procedure, and the total procedure time may remain the same.

Our study has several limitations. It was performed at a single institute with a small number of cases. Although the safety of M‐UEMR was suggested in this study, the frequency of adverse events in EMR and UEMR is low and should be evaluated in a larger number of patients for a more accurate evaluation. In addition, since this was a single‐arm study, comparison with conventional UEMR and a long‐term evaluation would be important. All mucosal defects were closed, and no adverse events occurred. This is our institution's strategy for managing lesions treated with electrocautery, including EMR and UEMR. However, the necessity of clipping after resection, including M‐UEMR, warrants further investigation. Furthermore, our primary aim was to assess whether M‐UEMR could be performed safely and yield outcomes comparable to those of conventional UEMR. Ideally, a non‐inferiority trial focusing on safety outcomes such as delayed bleeding, perforation, or post‐polypectomy syndrome would be appropriate. However, due to the low incidence of such adverse events in calculating an adequate sample size, we designed this study based on the expected R0 resection rate of M‐UEMR. Therefore, we set the R0 resection rate as the primary endpoint and designated safety as the first secondary endpoint. This design choice represents one of the limitations of our study, and future large‐scale comparative trials would be warranted to further evaluate the safety profile of M‐UEMR.

In conclusion, M‐UEMR demonstrated a high R0 resection rate for colorectal polyps with a safe profile. M‐UEMR is expected to be utilized as an alternative technique to UEMR for colorectal polyps. If a difficult situation is encountered, keeping a clear endoscopic view underwater during UEMR, due to dirty fluid inflow or insufficient water pooling after grasping the polyp, or concern for immediate bleeding, it can be beneficial to switch to M‐UEMR.

## Author Contributions

**Conception and design**: Kaizo Kagemoto, Koichi Okamoto, and Yoji Takeuchi; **Acquisition of data**: Kaizo Kagemoto, Koichi Okamoto, Motoko Sei, Shota Fujimoto, Ryo Shinomiya, Takeshi Mitsuhashi, Takanori Yoshimoto, Reiko Yokoyama, Tomoyuki Kawaguchi, Yoshifumi Kida, and Yasuhiro Mitsui; **Drafting the article**: Kaizo Kagemoto and Koichi Okamoto; **Analysis and interpretation of data**: Kaizo Kagemoto, Koichi Okamoto, and Yasuyuki Okada; **Critically revising the article**: Yutaka Kawano, Masahiro Sogabe, Hiroshi Miyamoto, and Yasushi Sato; **Statistical analysis**: Kaizo Kagemoto and Yasuyuki Okada; **Administrative support and study supervision**: Koichi Okamoto, Yoji Takeuchi, and Tetsuji Takayama.

## Conflicts of Interest

Author Tetsuji Takayama received a research grant from Fujifilm. Author Yoji Takeuchi is an Associate Editor of DEN Open: honoraria for lectures from Olympus, Fujifilm, Boston Scientific Japan, Daiichi‐Sankyo, Miyarisan Pharmaceutical, EA Pharma, Zeria Pharmaceutical, Viatris, Tsumura & CO, Kyorin Pharmaceutical, Otsuka Pharmaceutical Factory, Fuji Pharma Co, AI Medical Service, and Takeda Pharmaceuticals. The other authors declare no conflicts of interest.

## Funding

The authors have nothing to report.

## Ethics Statement

**Approval of the research protocol by an Institutional Reviewer Board**: The study protocol was approved by the ethics committee of Tokushima University Hospital.

## Consent

Informed consent was obtained from patients prior to study participation.

## Clinical Trial Registration

The trial was registered in the University Hospital Medical Information Network Clinical Trials Registry, UMIN 000048503.

## Supporting information




**VIDEO S1**: Modified underwater endoscopic mucosal resection for a colorectal polyp. A slightly elevated lesion in the transverse colon, a mostly regular pit pattern, deflating air and infusing water, a regular surface and vascular pattern, snaring underwater, removing the water and inflating with CO_2_, resection under CO_2_ insufflation, no residual lesion, and clipping easily.
